# Electroacupuncture Reduces Fibromyalgia Pain by Attenuating the HMGB1, S100B, and TRPV1 Signalling Pathways in the Mouse Brain

**DOI:** 10.1155/2022/2242074

**Published:** 2022-03-15

**Authors:** I-Han Hsiao, Yi-Wen Lin

**Affiliations:** ^1^College of Chinese Medicine, Graduate Institute of Acupuncture Science, China Medical University, Taichung 40402, Taiwan; ^2^Department of Neurosurgery, China Medical University Hospital, Taichung 404332, Taiwan; ^3^Chinese Medicine Research Center, China Medical University, Taichung 40402, Taiwan

## Abstract

Fibromyalgia is characterized by chronic and persistent widespread pain and generalized muscle tenderness, and it is refractory to treatment. The central nervous system (CNS) plays an important role, pain signalling, in fibromyalgia subjects. Electroacupuncture (EA) has been practiced for thousand years to treat many diseases that involve pain. We established fibromyalgia-like pain in mice using intermittent cold stress and investigated therapeutic effects and modes of action with EA. EA of 2 Hz and 1 mA was performed for 20 min at the ST36 acupoint in mice from Day 3 to Day 5. Our results showed that mechanical and thermal hyperalgesia were induced by intermittent cold stress (Day 5: mechanical: 1.43 ± 0.34 g; thermal: 3.98 ± 0.73 s) and were subsequently reversed by EA (Day 5: mechanical: 4.62 ± 0.48 g; thermal: 7.68 ± 0.68 s) or *Trpv1*^−/−^ (Day 5: mechanical: 4.38 ± 0.51 g; thermal: 7.48 ± 0.98 s). Activity in the HMGB1, S100B, and TRPV1 pathways was increased in the mouse prefrontal cortex, somatosensory cortex, thalamus, and amygdala with the stress treatment. This increase was attenuated by EA or *Trpv1*^−/−^. These results suggest potential targets for the treatment of TRPV1-dependant fibromyalgia pain.

## 1. Introduction

Fibromyalgia, also called fibromyalgia syndrome, is characterized by chronic widespread pain, severe fatigue, and sleep disturbances. Fibromyalgia patients can also develop somatic hypersensitivity to external stimuli, dysfunction of memory, or cognition and psychiatric disorders [1]. Fibromyalgia syndrome is one of the most frequent musculoskeletal problems with a prevalence that increases with age to 2%–8% worldwide [2]. To diagnose fibromyalgia, symptoms are evaluated and the proportions of the diagnostic criteria present at the time are assessed. The diagnostic criteria and consequently the treatment strategies have been constantly evolving [3–5]. There are several hypotheses regarding the causes of fibromyalgia such as a genetic predisposition, external environment stress, and peripheral (inflammatory) and central (cognitive-emotional) mechanisms that interplay and create dysperception of pain [1]. Because of its multiple etiologies, fibromyalgia necessitates different therapeutic approaches. Pharmacotherapy includes antidepressant, anticonvulsant, muscle relaxant, analgesic, and hypnotic drugs as well as cannabis [6], whereas nonpharmacological treatments comprise acupuncture, fitness [7], and patient education [8]. Most patients need more than two kinds of drugs. Moreover, opioids have limited efficacy in fibromyalgia treatment [1]. Several methods have been used recently to generate fibromyalgia-like pain rodent models [9]; that is, repeated muscle insult, biogenic amine depletion, and stress. Neyama et al. developed a mouse fibromyalgia model that nearly mimicked fibromyalgia patient behaviors and did not respond to opioid drugs [10].

Transient receptor potential vanilloid 1 (TRPV1) is present on the membranes of neurons and glial cells in the central and peripheral nervous systems. TRPV1 is a calcium channel mediating pain sensation. TRPV1 is activated by mechanical and thermal stimuli or by acidic conditions [11]. High-mobility group box 1 (HMGB1) was initially described as a nuclear factor enhancing transcription and was associated with infection, injury, and inflammation. HMGB1 is now considered to be a cytokine released from necrotic cells or secreted by immunocompetent cells activated by interleukin-1 (IL-1), interferon-*γ* (IFN-*γ*), tumor necrosis factor *α* (TNF-*α*), or endotoxin [12]. It targets cell surface receptors, such as receptor for advanced glycation end-products (RAGE), Toll-like receptor 2 (TLR2), and TLR4, to activate cytoplasmic signalling cascades that result in the modulation of DNA transcription by nuclear factor-*κ*B (NF-*κ*B). Recently, more attention has been paid to HMGB1 because of its contribution to CNS trauma injury [13]. The S100 family is a group of calcium-binding proteins characterized by their intra- or extracellular modulation of processes involved in tissue damage or cancer [14]. Furthermore, TRPV1 is reported as an inflammatory detector in cold stress pain. Cold stress augmented inflammatory modulators, such as HMGB1 and S100B, contribute to the pain process by activating TLR4 and RAGE. Cold stress also amplifies the expression of the PI3K-Akt-mTOR and pNF-*κ*B signalling pathways [15].

Acupuncture is a Chinese traditional medicine that has been used for more than 3,000 years. Its therapeutic use, especially for pain, has been approved by the World Health Organization. Traditional acupuncture is also called manual acupuncture (MA). Its manoeuvres include lifting, thrusting, rotating, and other similar techniques. In recent years, electrical current has been used in acupuncture to better standardize the experimental procedure and improve data reproducibility [16]. What the actual evidence-based mechanisms of EA are remains a mystery. The ST36 acupoint is commonly used in traditional Chinese medicine for the treatment of various pathological conditions, most notably pain [17–20]. EA has been shown to reduce inflammatory [17, 19], neuropathic [21], and fibromyalgia [18] pain in mice by promoting the release of endogenous opiates, dopamine, and adenosine.

The current study aimed to examine the effect of EA on cold stress-induced fibromyalgia pain in mice. Our previous work showed that EA attenuates complete Freund's adjuvant (CFA)-induced local inflammatory pain [17] and intermittent cold stress (ICS)-induced widespread pain [18]. We hypothesized that mutual regulation of the HMGB1/S100B and RAGE/TLR2/TLR4 axes and TRPV1 occurred but the mechanisms remain unclear. The present study aimed to investigate whether EA reduces fibromyalgia pain by inhibiting the HMGB1, S100B, and TRPV1 signalling pathways in the mouse brain. We hypothesized that EA attenuates fibromyalgia-like hyperalgesia by acting on TRPV1 in the mice brain. Our results provided evidence that TRPV1 might be a potential target for developing effective pain treatments.

## 2. Methods

### 2.1. Experimental Animals and Ethical Considerations

All mice were treated in accordance with the National Institute of Health Guide for the Care and Use of Laboratory Animals. The study protocol was approved by the Ethics Committee of the China Medical University, Taichung, Taiwan (permit no. CMUIACUC-2021-012). All C57BL/6 female mice weighing approximately 18–23 g and aged 8–12 weeks were used in this study. Totally, 36 female mice were used, including 27 wild-type (WT) mice (BioLASCO Taiwan Co., Ltd.) and 9 *Trpv1*^−/−^ mice (Jackson Lab, Bar Harbor, ME). A sample size of nine animals per group was calculated as the number required for an alpha of 0.05 and a power of 80%. In addition, the number of animals used here and their suffering were minimized. Mice were placed in Plexiglas cages and housed in a 12 : 12-hour light-dark cycle (8.00 a.m.–8.00 p.m.) room under temperature (25 ± 2°C) and relative humidity (60 ± 5%) controlling. All WT mice were randomly subdivided into 3 groups: control (Con) group, ICS group, and ICS with electroacupuncture (ICS + EA) group. The final group is TRPV1 knockout mice, which were exposed to ICS without treatment (ICS + KO). To conform with the 3Rs Principle of Humane Experimental Technique, we do not set the sham group (ICS + Sham) according to previous articles.

### 2.2. Fibromyalgia Model

The fibromyalgia-like pain model was initiated as previously described (Nishiyori and Ueda, 2008). Except the control group, all mice were treated with ICS procedure. Firstly, 2 mice were put together in a Plexiglas cage (13 × 18.8 × 29.5 cm). On the first day, named Day 0, the mice were housed in a cold room at 4°C overnight (starting from 4:30 p.m.–10 a.m.). Then, at 10 a.m. on Day 1, mice were transferred to a normal temperature 24°C room for 30 min. After that, mice were returned back to the cold room again for 30 min. These processes were repeated for a period of 6.5 hours (10 a.m.–4:30 p.m.). Finally, mice were placed in the 4°C cold room again overnight, from 4:30 p.m. of Day 1 to 10 a.m. of Day 2. The procedure of alternation of room temperatures amid 4°C and 24°C every 30 min during 10 a.m.–4:30 p.m. was repeated two times daily, on Day 1 and Day 2. At 10 a.m. on Day 3, the mice were put back to the 24°C room again. Control group mice (*n* = 9) were constantly kept at room temperature of 24°C from Day 0 to Day 3, without any interventions applied. The nociception testing was applied to all 4 groups at the same time periods, that is, before the beginning of the ICS procedure on Day 0 and on Day 5 before sacrificing.

### 2.3. EA Treatment

EA treatment was performed in the ICS + EA group (*n* = 8). During this procedure, fully inhalation anesthesia was performed using 5% isoflurane gas for induction and 1% isoflurane gas for maintenance. All mice were anesthetized with isoflurane at the same period to reduce the anesthetic effect. A pair of stainless steel acupuncture needles (1 inch, 36G; YU KUANG, Taiwan) was inserted bilaterally into the ST36 acupoint. The ST36 acupoint was chosen as it is commonly used in traditional Chinese medicine for the treatment of various pathological conditions, most notably pain. The ST36 acupoint in mice is located approximately 3–4 mm below and 1–2 mm lateral to the midpoint of the knee. The needles were perpendicularly inserted to a depth of 3–4 mm. The electric stimulation was delivered using a Trio 300 stimulator (Ito, Japan) at an intensity of 1 mA for 20 min at 2 Hz with a pulse width of 100 *μ*s. EA was performed from Day 3 to Day 5.

### 2.4. Nociceptive Behavior Test

These behavior tests consist of the von Frey test (mechanical hyperalgesia) and Hargreaves test (thermal pain). All groups received behavior tests at two time points: one hour before sending mice into the cold room on Day 0 and one hour after EA treatment on Day 5. Both tests were performed in the behavior analysis room under temperature 24°C. Mice were placed into a metal mesh (75 × 25 × 45 cm), and each one was covered with a Plexiglas cage (10 × 6 × 11 cm) and acclimated for 30 min to 1 hour to let them calm, but not sleeping or grooming. Mechanical sensitivity was measured by testing the force of responses to stimulation with 3 applications of the electronic, calibrated von Frey filament (IITC Life Science Inc., USA). The plantar region of the right hind paw was tested by the tip of the filament and mechanical stimulation. Second, the Hargreaves assessment was conducted. Mice were moved to the top of a glass sheet, and each one was covered by a Plexiglas cage and acclimated at least for 30 min. Thermal pain was measured by testing the time of responses to thermal stimulation with 3 applications using a Hargreaves test IITC analgesiometer (IITC Life Sciences; SERIES8, Model 390G). The thermal stimulator was placed under the glass sheet, and the focus of the projection bulb was aimed exactly at the middle of the plantar surface of the right hind paw. The nociception threshold was assessed using the latency of paw withdrawal upon stimuli. A cut-off time of 20 s was set to prevent tissue damage.

### 2.5. Immunoblotting

After the completion of the final behavior test on Day 5, the mice were sacrificed via anaesthetizing with 1% isoflurane inhalation. The prefrontal cortex (PFC), somatosensory cortex (SSC), thalamus, and amygdala tissues were immediately excised to extract proteins for western blot. Tissues were initially placed on ice and later stored at −80°C, pending protein extraction. Total proteins were homogenized in cold radioimmunoprecipitation (RIPA) lysis buffer containing 50 mM Tris-HCl pH 7.4, 250 mM NaCl, 1% NP-40, 5 mM EDTA, 50 mM NaF, 1 mM Na3VO4, 0.02% NaN3, and 1× protease inhibitor cocktail (AMRESCO). The extracted proteins were subjected to 8% SDS-Tris glycine gel electrophoresis and transferred to a PVDF membrane. The membrane was blocked with 5% nonfat milk in TBS-T buffer (10 mM Tris-HCl pH 7.5, 100 mM NaCl, 0.1% Tween-20) and then incubated with a primary antibody against TRPV1 (∼95 kDa, 1 : 1000; Alomone, Israel), HMGB1 (∼28 kDa, 1 : 1000; Alomone, Israel), S100B (∼10 kDa, 1 : 1000; Millipore, USA), TLR4 (∼35 kDa, 1 : 1000; Millipore, USA), RAGE (∼42 kDa, 1 : 1000; Millipore, USA), pPI3K (∼125 kDa, 1 : 1000; Millipore, USA), pAkt (∼60 kDa, 1 : 1000; Millipore, USA), pmTOR (∼60 kDa, 1 : 500; Millipore, USA), pERK1/2 (∼42–44 kDa, 1 : 1000; Millipore, USA), pp38 (∼42 kDa, 1 : 1000; Millipore, USA), pJNK (∼42 kDa, 1 : 1000; Millipore, USA), and pNF-*κ*B (∼65 kDa, 1 : 1000; Millipore, USA) in TBS-T with 1% bovine serum albumin. Peroxidase-conjugated anti-rabbit antibody, anti-mouse antibody, or anti-goat antibody (1 : 5000) was used as the appropriate secondary antibody. The bands were visualized by an enhanced chemiluminescent substrate kit (PIERCE) with LAS-3000 Fujifilm (Fuji Photo Film Co., Ltd.). Where applicable, the image intensities of specific bands were quantified with NIH Image J software (Bethesda, MD, USA).

### 2.6. Immunofluorescence

After the final behavior test, remainder euthanized with a 5% isoflurane via inhalation and intracardially perfused with normal saline followed by 4% paraformaldehyde. The brain was immediately dissected and postfixed with 4% paraformaldehyde at 4°C for 3 days. The tissues were placed in 30% sucrose for cryoprotection overnight at 4°C. The brain was embedded in an optimal cutting temperature (OCT) compound and rapidly frozen using liquid nitrogen before storing the tissues at −80°C. Frozen segments were cut at 20 *μ*m width on a cryostat and then instantaneously placed on glass slides. The samples were fixed with 4% paraformaldehyde and then incubated with a blocking solution, consisting of 3% BSA, 0.1% Triton X-100, and 0.02% sodium azide for 1 h at room temperature. After blocking, the samples were incubated with the primary antibody (1 : 200; Alomone), TLR2, and pNF-*κ*B, prepared in 1% bovine serum albumin solution at 4°C overnight. The samples were then incubated with the secondary antibody (1 : 500), 488-conjugated AffiniPure donkey anti-rabbit IgG (H + L), and peroxidase-conjugated AffiniPure donkey anti-mouse IgG (H + L) for 2 h at room temperature before being fixed with coverslips for immunofluorescence visualization. The samples were observed using an epifluorescence microscope (BX-51; Olympus, Japan) with 20× numerical aperture (NA = 1.4) objective.

### 2.7. Statistical Analysis

Statistical analysis was performed using the SPSS statistic program. All statistic data are presented as the mean ± standard error (SEM). The Shapiro–Wilk test was performed to test the normality of data. Statistical significance among all groups was tested using the repeated-measure ANOVA test, followed by post hoc Tukey's test. Values of *P* < 0.05were considered statistically significant.

## 3. Results

### 3.1. EA at the ST36 Acupoint or Trpv1^−/−^ Attenuated Nociceptive Behaviors in the Intermittent Cold Stress (ICS) Mouse Model

**Figure 1:**
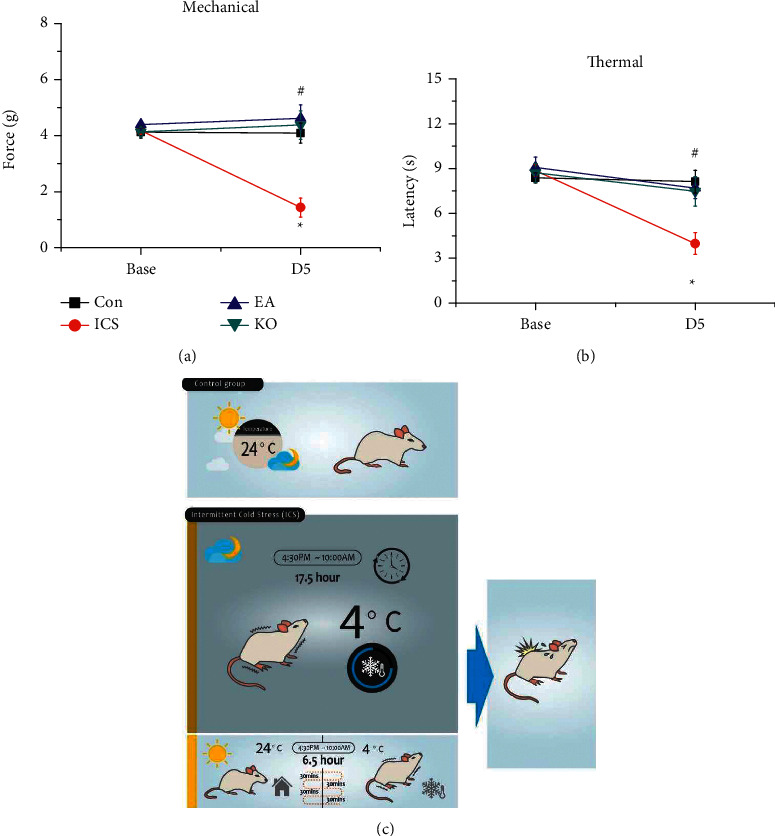
Comparative graph showing the hind paw withdrawal threshold and latency. Black: control group, red: ICS group, blue: ICS + electroacupuncture (EA) group, green: transient receptor vanilloid member one deletion (*Trpv1*^−/−^) group. ^*∗*^ indicates statistical significance with *p* < 0.05 when compared with the control group. ^#^ indicates statistical significance with *p* < 0.05 when compared with the ICS group. (a) Mechanical hyperalgesia measured by the von Frey test in the four groups. (b) Thermal hyperalgesia assessed by the Hargreaves test in the four groups. (c) Schematic illustration showing the ICS procedure. Control mice stayed in a 24°C environment day and night for five days. Mice subjected to ICS were kept in a 4°C environment for 17.5 hours (from 4:30 p.m. to 10:00 a.m.) from Day 0 to 3. Between 10:00 a.m. and 4:30 p.m. (total duration: 6.5 hours), they were placed in a room at 24°C. In this 6.5-hour period, the mice were subjected to intermittent environment temperature change (24°C and 4°C) for 30 min each time. The procedure was terminated on Day 3 at 10:00 a.m., and fibromyalgia-like pain was assessed.

We used von Frey filaments to evaluate mechanical hyperalgesia. At the baseline (i.e., Day 0, before ICS induction), there was no significant difference between the groups (Figure 1(a)). Five days later, the values for the control group had not changed as the mice were submitted to no intervention (Figure 1(a), black line). However, the ICS group (with induced fibromyalgia-like pain) showed mechanical hyperalgesia as the paw withdrawal threshold was decreased compared with that of the controls (Figure 1(a), red line, D5, ^*∗*^*P* < 0.05, 1.43 ± 0.34 g, *n* = 9). The mechanical hyperalgesia was significantly attenuated in mice that received EA treatment at the ST36 acupoint three times after ICS induction (EA group) compared with that of the ICS group (Figure 1(a), blue line, D5, ^#^*P* < 0.05, 4.62 ± 0.48 g, *n* = 9). This attenuation was similar in mice with *Trpv1*^−/−^ (Figure 1(a), green line, D5, ^#^*P* < 0.05 compared with control, 4.38 ± 0.51 g, *n* = 9). Thermal hyperalgesia was measured with the Hargreaves test, which evaluates the response (paw withdrawal latency) to a heat stimulus. The results were similar to those obtained for mechanical hyperalgesia as we measured significant thermal hyperalgesia after ICS induction (Figure 1(b), red line, D5, ^*∗*^*P* < 0.05, 3.98 ± 0.73 s, *n* = 9). This hyperalgesia was attenuated by EA (Figure 1(b), blue line, D5, ^#^*P* < 0.05, 7.68 ± 0.68 s, *n* = 9) or *Trpv1*^−/−^ (Figure 1(b), green line, D5, ^#^*P* < 0.05, 7.48 ± 0.98 s, *n* = 9). These data suggest that EA is an efficient treatment for fibromyalgia pain. Moreover, *Trpv1*^−/−^ mice have the effect.  Figure 1(c) illustrates the ICS protocol.

### 3.2. EA and Trpv1^−/−^ Reversed ICS-Induced Activation of Signalling Pathways in the Mice Prefrontal Cortex (PFC)

**Figure 2:**
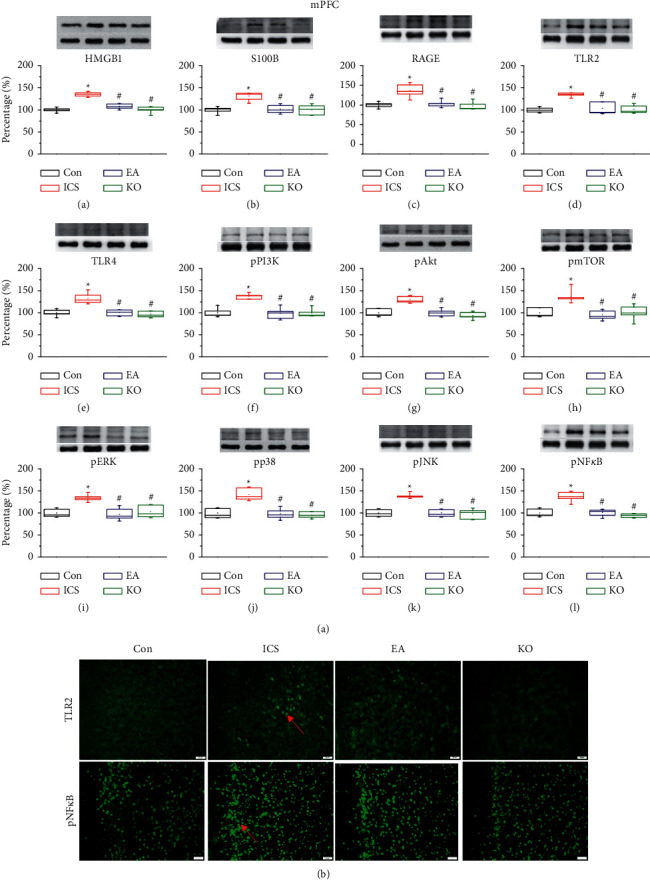
(a) Expression of extracellular neurotransmitters ((A) HMGB1, (B) S100B), receptors ((C) RAGE, (D) TLR2, (E) TLR4), and cytoplasmic inflammation signal molecules ((F) pPI3K, (G) pAkt, (H) pmTOR, (I) pERK, (J) pp38, (K) pJNK, (L) pNF-*κ*B) in the mouse PFC. The four lanes on the immunoblots correspond to the protein bands of, in order, control (Con), ICS-induced fibromyalgia (ICS), electroacupuncture (EA), and *Trpv1*^−/−^ groups. ^*∗*^ indicates statistical significance with *P* < 0.05 when compared with the Con group. # indicates statistical significance with *P* < 0.05 when compared with the ICS group. (b) Immunofluorescence staining of TLR2 and pNF-*κ*B in the mouse PFC. Immunopositive signals (light green, indicated by a red arrow) were detected for TLR2 and pNF-*κ*B. Scale bar: 100 *μ*m.

The PFC is connected to the thalamus, amygdala, and other basal nuclei and is important for pain processing. We used western blot and immunofluorescence staining to check whether ICS affects neural inflammatory mediators, i.e., HMGB1 and S100B, and signalling pathways in the PFC. HMGB1 and S100B expression in the PFC was higher in the ICS group than controls (Figure 2(a), A and B, red column, ^*∗*^*P* < 0.05, *n* = 6). This increase in HMGB1 and S100B levels was prevented in mice receiving EA at the ST36 acupoint (Figure 2(a), A and B, blue column, ^#^*P* < 0.05, *n* = 6) and in *Trpv1*^−/−^mice (Figure 2(a), A and B, green column, ^#^*P* < 0.05, *n* = 6). To further characterize the signalling pathways, the presence of three receptors, i.e., TLR2, TLR4, and RAGE, at the neuronal plasma membrane was evaluated. ICS induced a receptor overexpression (Figure 2(a), C–E, red column, ^*∗*^*P* < 0.05, *n* = 6), which was reversed by EA and *Trpv1*^−/−^ (Figure 2(a), C–E, blue and green columns, ^#^*P* < 0.05, *n* = 6). Similarly, EA and *Trpv1*^−/−^ reversed the ICS-induced increase of the pPI3K-pAkt-pmTOR axis, MAPKs (i.e., pERK, pp38, and pJNK) levels (Figure 2(a), F–K, ^#^*P* < 0.05, *n* = 6), and the transcriptional factor pNF-*κ*B (Figure 2(a), ^#^*P* < 0.05, *n* = 6). We also showed that CSP increased TLR2 and pNF-*κ*B expression (Figure 2(b)). This overexpression was reduced by EA and *Trpv1*^−/−^ (Figure 2(b), ^#^*P* < 0.05, *n* = 3).

### 3.3. Similar Effects of EA at the ST36 Acupoint and Trpv1^−/−^ on Protein Expression in the Layers II/III of the Somatosensory Cortex (SSC) of Mice Subjected to ICS

**Figure 3:**
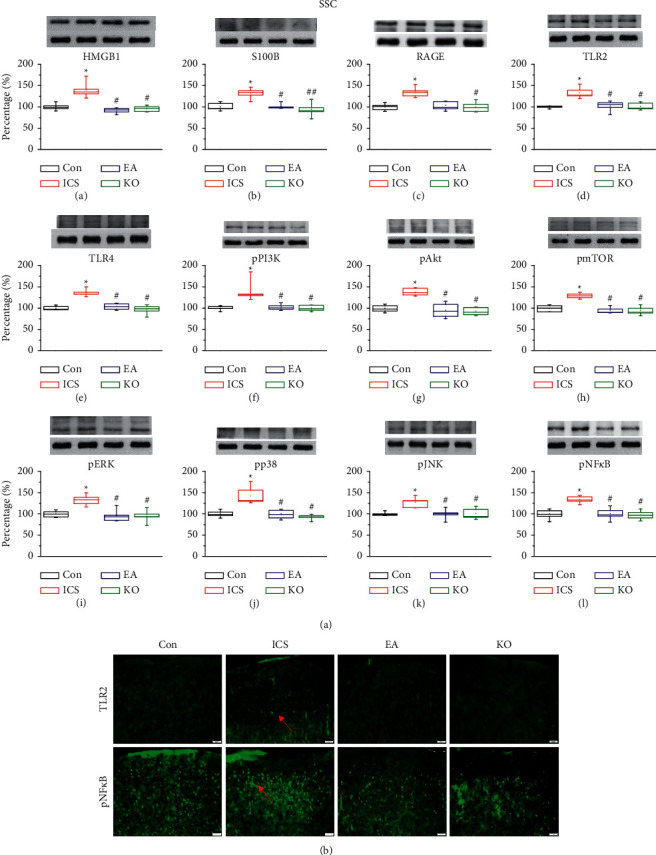
(a) Expression of extracellular neurotransmitters ((A) HMGB1, (B) S100B), receptors ((C) RAGE, (D) TLR2, (E) TLR4), and cytoplasmic inflammation signal molecules ((F) pPI3K, (G) pAkt, (H) pmTOR, (I) pERK, (J) pp38, (K) pJNK, (L) pNF-*κ*B) in the mouse SSC. The four lanes on the immunoblots correspond to the protein bands of, in order, control (Con), ICS-induced fibromyalgia (ICS), electroacupuncture (EA), and *Trpv1*^−/−^ groups. ^*∗*^indicates statistical significance with *P* < 0.05 when compared with the Con group. ^#^ indicates *P* < 0.05 statistical significance when compared with the ICS group. (b) Immunofluorescence staining of TLR2 and pNF-*κ*B in the mouse SSC. Immunopositive signals (light green, indicated by a red arrow) were detected for TLR2 and pNF-*κ*B. Scale bar: 100 *μ*m.

Recent findings evidenced a role of the somatosensory cortex in the nociceptive transmission pathway. Western blot analysis of SSC samples showed significantly higher expression of extracellular transmitters and cell membrane receptors in ICS mice (Figure 3(a), A–E, red column, ^*∗*^*P* < 0.05, *n* = 6). We also found an increased activation of signalling pathways in the cytoplasm (Figure 3(a), F–L, red column, ^#^*P* < 0.05, *n* = 6). EA at ST36 and *Trpv1*^−/−^ attenuated the effects of ICS on protein expression (Figure 3(a), A–-L, blue and green columns, ^#^*P* < 0.05, *n* = 6) and had very similar effects on each protein. Immunofluorescence staining in the SSC of ICS mice was increased compared with those of mice subjected to EA or *Trpv1*^−/−^ (Figure 3(b)).

### 3.4. EA at the ST36 Acupoint and Trpv1^−/−^ Significantly Attenuated the Increase of Neurotransmitter and Downstream Molecule Levels in the Thalamus of ICS Mice

**Figure 4:**
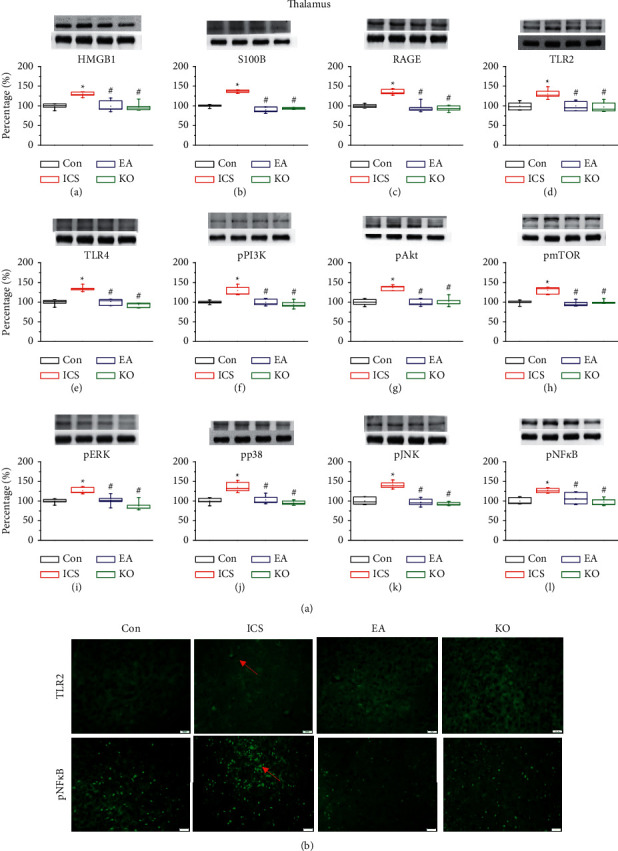
(a) Expression of extracellular neurotransmitters (A) HMGB1, (B) S100B), receptors (C) RAGE, (D) TLR2, (E) TLR4) and cytoplasmic inflammation signal molecules (F) pPI3K, (G) pAkt, (H) pmTOR, (I) pERK, (J) pp38, (K) pJNK, (L) pNF-*κ*B) in the mouse thalamus. The four lanes on the immunoblots correspond to the protein bands of, in order, control (Con), ICS-induced fibromyalgia (ICS), electroacupuncture (EA) and *Trpv1*^−/−^ groups. ^*∗*^ indicates statistical significance with *P* < 0.05 when compared with the Con group. # indicates statistical significance with *P* < 0.05 when compared to the ICS group. (b) Immunofluorescence staining of TLR2 and pNF-*κ*B in the mouse thalamus. Immunopositive signals (light green, indicated by a red arrow) were detected for TLR2 and pNF-*κ*B. Scale bar: 100 *μ*m.

Thalamic nuclei affect the motivational components of pain and are involved in sensory discrimination. S100B and HMGB1 are secreted by non-neuronal cells in the brain, such as astrocytes or microglial cells. Western blot analysis revealed an increased protein density in the thalamus of mice subjected to ICS (Figure 4(a), A and B, red column, ^*∗*^*P* < 0.05, *n* = 6), whereas EA at ST36 and *Trpv1*^−/−^ groups reversed this effect (Figure 4(a), A and B, blue and green columns, ^#^*P* < 0.05, *n* = 6). The amount of receptors RAGE, TLR2, and TLR4 were also increased in the ICS-induced pain group (Figure 4(a), C–E, red column, ^*∗*^*P* < 0.05, *n* = 6). EA at ST36 and *Trpv1*^−/−^ reversed ICS effects (Figure 4(a), C–E, blue and green columns, ^#^*P* < 0.05, *n* = 6). This reversion was also observed in the signalling pathways triggered by the receptors (Figure 4(a), F–L, ^#^*P* < 0.05, *n* = 6). Immunofluorescence analyses (Figure 4(b)) showed that the EA at ST36 and *Trpv1*^−/−^ abolished the pNF-*κ*B increase observed in the ICS-induced pain group.

### 3.5. EA Reversed the Increase of the Levels of Inflammatory Signalling Pathway Mediators Induced by ICS in the Amygdala

**Figure 5:**
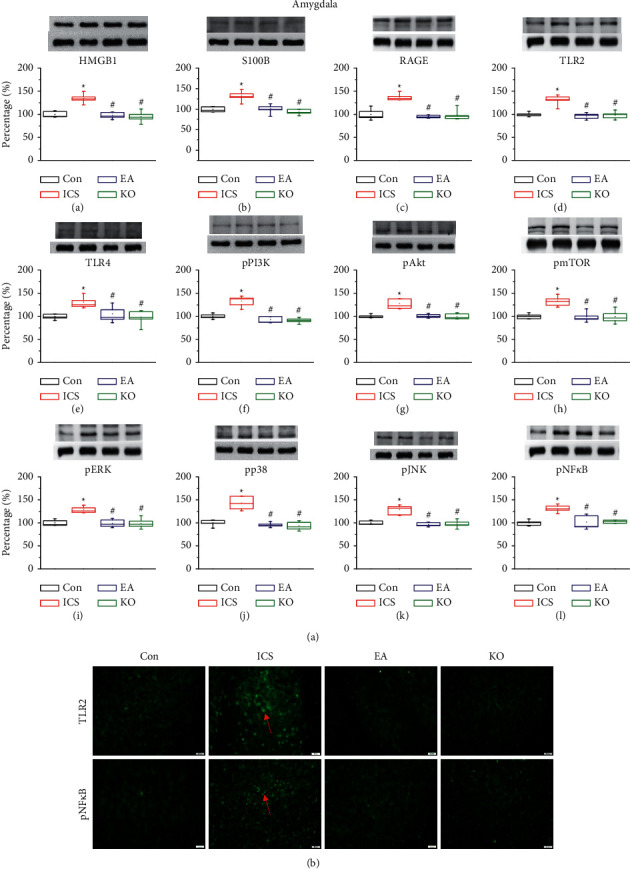
(a) The expression of extracellular neurotransmitters ((A) HMGB1, (B) S100B), receptors ((C) RAGE, (D) TLR2, (E) TLR4), and cytoplasmic inflammation signal molecules ((F) pPI3K, (G) pAkt, (H) pmTOR, (I) pERK, (J) pp38, (K) pJNK, (L) pNF-*κ*B) in the mouse amygdala. The four lanes on the immunoblots correspond to the protein bands of, in order, control (Con), ICS-induced fibromyalgia (ICS), electroacupuncture (EA), and *Trpv1*^−/−^ groups. ^*∗*^ indicates statistical significance with *P* < 0.05 when compared with the Con group. # indicates statistical significance with *P* < 0.05 when compared with the ICS group. (b) Immunofluorescence staining of TLR2 and pNF-*κ*B in the mouse amygdala. Immunopositive signals (light green, indicated by a red arrow) were detected for TLR2 and pNF-*κ*B. Scale bar: 100 *μ*m.

Amygdala is important for pain modulation and advanced emotional-affective dimension of pain. After ICS induction, amygdala samples were collected to measure protein levels. ICS significantly increased the levels of HMGB1, S100B, TLR2, TLR4, and RAGE (Figure 5(a), A–E, red column, ^*∗*^*P* < 0.05, *n* = 6). EA and *Trpv1*^−/−^ reversed this overexpression (Figure 5(a), A–E, blue and green columns, ^#^*P* < 0.05, *n* = 6) similarly as in the PFC, SSC, and thalamus. Furthermore, the levels of downstream molecules such as pPI3K, pAkt, and pmTOR (Figure 5(a), F–H, red column, ^*∗*^*P* < 0.05, *n* = 6) were higher in the amygdala of the ICS group than those measured in the EA and *Trpv1*^−/−^ groups (Figure 5(a), F–H, blue and green columns, ^#^*P* < 0.05, *n* = 6). Regarding the MAPK signalling pathway, pERK, pp38, and pJNK levels were significantly higher in the thalamus of the ICS group than that of the control group (Figure 5(a), I–K, red column, ^*∗*^*P* < 0.05, *n* = 6) and in the EA and *Trpv1*^−/−^ groups (Figure 5(a), I–K, blue and green columns, ^#^*P* < 0.05, *n* = 6). The ICS group also presented higher pNF-*κ*B levels than the control group (Figure 5(a), L, red column, ^*∗*^*P* < 0.05, *n* = 6), whereas EA and *Trpv1*^−/−^ groups prevented this increase (Figure 5(a), L, blue and green columns, ^#^*P* < 0.05, *n* = 6). Immunofluorescence staining of TLR2 and pNF-*κ*B (Figure 5(b)) was largely decreased in EA and *Trpv1*^−/−^ animals compared with those in the ICS group.

## 4. Discussion

**Figure 6:**
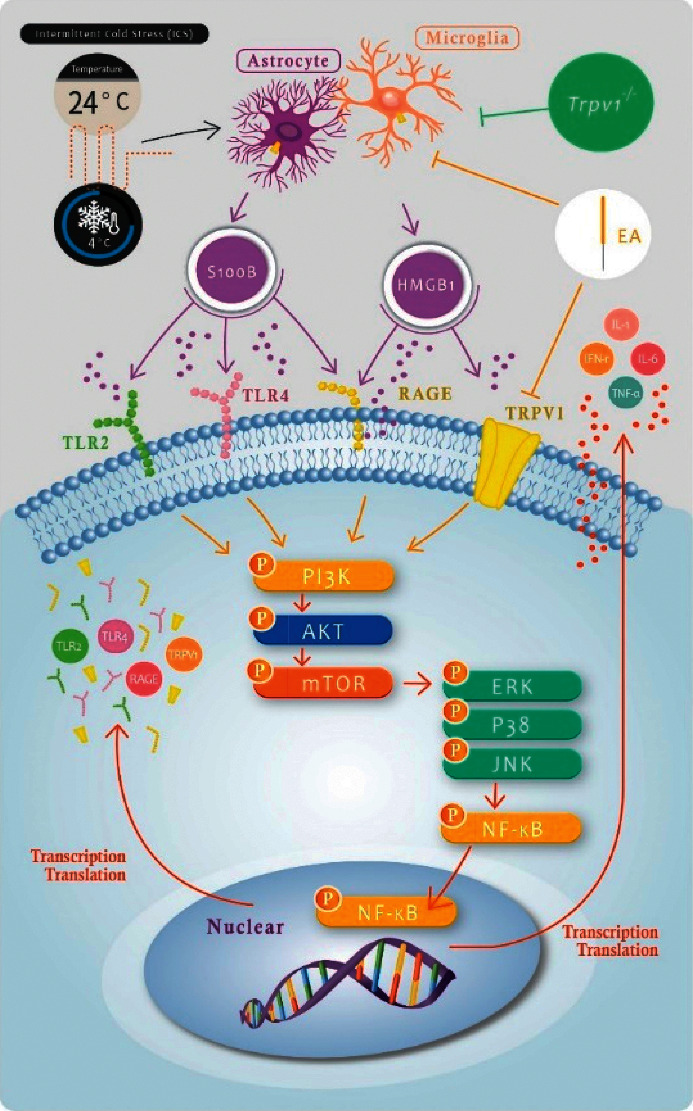
Schematic illustration of neuronal and non-neuronal mechanisms underlying EA-mediated analgesic effect on ICS-induced fibromyalgia pain. The summary diagram shows the importance of and mechanisms involving glial cells (astrocytes and microglia) and TRPV1 in fibromyalgia pain. EA inhibits HMGB1 and S100B release from non-neuronal cells or directly inhibits TRPV1 on the plasma membrane. Mice with a TRPV1 gene deletion (*Trpv1*^−/−^) have the same phenotype than mice treated with EA.

The results are summarized in a schematic illustration in Figure 6. When mice are subjected to ICS, astrocytes and microglial cells secrete abundant amounts of HMGB1 and S100B. These neurotransmitters bind to the TLR2, TLR4, RAGE, and TRPV1 receptors on the cytoplasmic membrane. Subsequently, these receptors activate cytoplasmic signalling pathways, namely the pPI3K-pAkt-pmTOR and MAPK (composed of pERK, pp38, and pJNK) pathways. MAPK signalling activates the transcription factor pNF-*κ*B, resulting in the production of more inflammatory factors and receptors [22]. This pathway activation in the mouse brain is responsible for hyperalgesia behavior. Our behavioral, western blot, and immunostaining analyses revealed that EA at the ST36 acupoint and *Trpv1*^−/−^ had similar effects in preventing the increased protein expression induced by ICS. Our recent results suggested that TRPV1, which is expressed on neuronal and non-neuronal cell membranes, is the main receptor modulating the fibromyalgia pain signalling pathway. EA at ST36 might relieve fibromyalgia pain by directly inhibiting neuronal TRPV1 or TRPV1 on glial cells to interrupt the signalling pathway [15]. In this study, we found that activity in the HMGB1, S100B, and TRPV1 pathways was increased in the mouse prefrontal cortex, somatosensory cortex, thalamus, and amygdala. The increase was attenuated by EA or *Trpv1*^−/−^.

Fibromyalgia not only affects individuals but is also a substantial societal burden. Significant costs and loss of productivity related to fibromyalgia have been reported. Berger et al. used a United States health-insurance claim database and found that the mean of annual direct medical costs for fibromyalgia patients reached US$9,573 with a US$20,135 standard deviation [23]. Lacasse et al. collected data from 57 fibromyalgia patients and showed that an average of 5.59 days of work was lost during a 3-month period due to pain, which was equal to 3.19 weeks annually [24]. Therefore, albeit challenging, finding a low-cost and effective therapy that could alleviate the burden of fibromyalgia on patients and society is essential. A good animal model is a valuable investigation tool in basic pain research. For example, local inflammation pain is induced by compete Freund's adjuvant (CFA) injection and local neuropathic pain is induced by spared nerve injury or a chronic constrict injury model. However, fibromyalgia is a complex syndrome with chronic pain affecting the whole body and it is resistant to opioid drugs or nonsteroidal anti-inflammatory drugs [10]. Therefore, a fibromyalgia animal model is difficult to generate. Nishiyori et al. published an ideal fibromyalgia model after a series of studies [25]. They modified previous work and exposed rodents to a 4°C environment. They showed that ICS, which consists of alternations between environments at 4°C and at 24°C (room temperature), induced long-term fibromyalgia-like pain compared with what was observed with continued cold stress. In addition to long-lasting whole-body pain (mechanical allodynia lasts 12 days and thermal hyperalgesia lasts 15 days), the response to drugs in the ICS model is almost the same as responses in fibromyalgia patients. Indeed, gabapentin had a complete antiallodynia action, whereas morphine displayed no analgesia effects [10]. Fibromyalgia patients also present with characteristic emotional problems. Our previous study found that rodents subjected to the ICS procedure for six weeks showed depression symptoms similar to human clinical presentations. Therefore, the ICS model is ideal and reliable for fibromyalgia studies [26].

Acupuncture or so-called MA is a traditional Chinese medicine that is used for the treatment of physical problems by modulating Qi along meridians. It is especially efficient against pain. In recent decades, peripheral electrical stimulation has been used to treat various diseases. For example, in transcutaneous electrical nerve stimulation (TENS), electrodes are placed on the skin to relieve neuropathic and chronic pain [27, 28]. In EA, probes are inserted through the skin into the tissue at acupoints, which have lower electoral resistance, and damage vessels or nerves are avoided. According to a series of publications, low-frequency current (2–4 Hz) stimulations result in an analgesia effect by stimulating the secretion of the opioid peptide enkephalin and activating the *μ*-opioid receptors in the central nerve system, whereas higher frequencies (100–200 Hz) did not provoke the same response [16].

Caterina et al. reported that TRPV1 is a calcium ion channel and that capsaicin is its main agonist. It was found that capsaicin induces a burning pain sensation by selectively activating sensory neurons that transmit noxious stimuli to the CNS [11]. In 2014, Wu et al. showed that TRPV1 is also a mechanosensitive channel that is abundantly present in neural and non-neural cells of the ST36 acupoint. Traditional MA has been used on CFA-induced local inflammation pain models and successfully attenuated the rodent pain behavior, thanks to mechanical manoeuvres that regulated propagation of the calcium wave [29]. Since that report, TRPV1 has been considered to be an “acupuncture-responding channel.” Lu et al. proposed MA to be replaced by EA for pain attenuation study using TRPV1, which would improve the scientific method as EA is more easily quantified and controlled [30]. In 2017, Liao et al. investigated the details of molecular cascades triggered by EA to attenuate inflammatory pain through the TRPV1 signalling pathway and proposed the following mechanism. Glial cells (astrocytes and microglial cells) around neurons have TRPV1 receptors, and EA directly inhibits the glial TRPV1 receptors to prevent S100B secretion and consequently S100B binding to RAGE on the neuronal membrane. This results in the inhibition of inflammatory processes. They also suggested that EA directly inhibits neuronal TRPV1s and inactivates the downregulating signalling pathways associated with the expression of Nav1.7 and Nav1.8. They further showed that EA triggers endomorphin and adenosine release to attenuate inflammatory pain. EA has been successfully used to inhibit the activation of TRPV1 in numerous nociceptive models [17]. We concluded that pain linked to CFA-induced local inflammation is TRPV1-dependant pain.

In 2020, Hsu et al. generated another fibromyalgia murine model by injecting rodents with acid saline. They use EA at the ST36 acupoint to successfully improve fibromyalgia-like pain behavior. Western blot analyses and immunohistochemical staining of brains showed that downregulation of the signalling factors, ranging from TRPV1 to pERK pathway members, was inhibited in the rodent brain after EA [31]. A recent article showed that the symptoms of fibromyalgia-like pain induced by ICS were improved when the TRPV1 gene was deleted (*Trpv1*^−/−^). These data suggest that EA might directly inhibit TRPV1 on neuronal membranes and its downstream pathways [18]. The transmission of pain sensations is mediated mainly by the amygdala, thalamus, PFC, and somatosensory cortex in the mouse brain. Under chronic pain conditions, the amygdala receives the nociceptive signal from the brain stem and transfers it to the thalamus [32]. The nociceptive signal is sent from the thalamus to higher brain centers, namely the prefrontal and somatosensory cortices, to produce an advanced response [33]. In our study, the mice suffered from ICS and the brain received signals from the peripheral nerves. S100B and HMGB1 were abundantly secreted by the microglia and astrocytes in the brain. These two proteins bound the RAGE, TLR2, and TLR4 and activated downstream pathways from pPI3K to pNF-*κ*B. Activated NF-*κ*B (pNF-*κ*B) entered the nucleus and bound to DNA to stimulate the transcription of additional inflammatory proteins. There are many more receptors and neuronal proteins that are involved in inflammation, such as IL-1, IL-6, IFN-*γ*, and TNF-*α*. These factors mediate positive affective states and hypersensitivity to pain as well as allodynia and hyperalgesia development [34, 35]. Our previous study suggested that EA has an analgesic effect associated with TRPV1 downregulation in the mouse hypothalamus, PAG, CVI, and CVII brain areas [15]. We demonstrated that inflammatory mediators can modulate the TRPV1 signalling pathway for managing fibromyalgia pain. We also implicated TRPV1 and related components in responses to fibromyalgia-like pain in the ICS model in the prefrontal cortex, somatosensory cortex, hippocampus, and thalamus of the brain [18]. We also demonstrated that TRPV1 proteins and related molecules were meaningfully reduced in the mice mPFC, hippocampus, and PAG. Attenuation of TRPV1 and its related molecules implicated their involvement in the pathogenesis of chronic pain and comorbidity of depression [26]. In this study, we indicated that the HMGB1, S100B, and TRPV1 pathways were increased in the mouse medical prefrontal cortex, somatosensory cortex, thalamus, and amygdala.

In conclusion, fibromyalgia is a syndrome that requires implementation of multiple strategies for treatment. Until now, acupuncture was not among the dominant therapeutic options for fibromyalgia. The present work provides strong evidence in favor of acupuncture as a treatment for fibromyalgia. Moreover, acupuncture might allow use of lower drug dosages and reduction of additional treatment. Finally, we showed that S100B and HMGB1 regulate TRPV1 and its signalling pathways and propose TRPV1 as a potential therapeutic target for fibromyalgia.

## Data Availability

The data used to support the findings of this study are available from the corresponding author upon request.
